# Orchestration of PKC‐Mediated Inhibition and Calcium Release‐Mediated Activation of BK Currents in Rat Vascular Smooth Muscle Cells by Melatonin Confers a BK‐Channel‐Dependent Restraint on Melatonin‐Induced Vasocontraction

**DOI:** 10.1096/fj.202601214R

**Published:** 2026-05-12

**Authors:** Anastasia Pyanova, Vjatscheslav U. Kalentchuk, Vladimir N. Serebryakov, Rudolf Schubert

**Affiliations:** ^1^ Physiology, Institute of Theoretical Medicine, Faculty of Medicine University of Augsburg Augsburg Germany; ^2^ Moscow State University, Department of Pathophysiology Moscow Russia; ^3^ Institute of Experimental Cardiology Cardiology Research Center Moscow Russia

**Keywords:** arteries, BK channel, IP_3_, melatonin, PKC, smooth muscle

## Abstract

Vascular smooth muscle BK channels are affected by several signaling pathways, in particular by PKC and calcium release from intracellular stores. However, little is known about how the cross‐talk of these pathways is orchestrated. Melatonin is widely recognized for its role in circadian regulation, yet its vascular actions remain incompletely understood. The hypothesis that cross‐talk of PLC‐mediated stimulation of PKC and calcium release determines the role of the BK channel in melatonin‐induced vasocontraction was tested. The patch‐clamp technique was used on freshly isolated smooth muscle cells and wire myography on intact rat tail arteries. Melatonin markedly stimulates an outward current. Iberiotoxin, a specific BK channel inhibitor, abolished this effect. The effect of melatonin was not affected by 4P‐PDOT, a selective MT_2_–receptor antagonist, but considerably depressed by luzindole, a MT_1_‐ and MT_2_–receptor antagonist. Mechanistically, the melatonin‐induced stimulation of the BK current was abolished by GDPβS, an inhibitor of G‐protein function, U‐73122, a PLC‐inhibitor, and low molecular weight heparin, an inhibitor of IP_3_ –induced calcium release. Calphostin C, a specific PKC‐inhibitor, further stimulated the BK current in the presence of melatonin, suggesting functional cross‐talk between distinct PLC‐dependent signaling branches. At the functional level, melatonin reversibly contracted intact vessel preparations. Vessel pre‐treatment with TEA or iberiotoxin augmented the effect of melatonin. Together, these findings identify BK channels as a key downstream target of melatonin signaling in vascular smooth muscle and reveal a previously unrecognized PLC‐dependent signaling cross‐talk that contributes to melatonin‐induced vasocontraction.

## Graphical abstract

Melatonin‐evoked BK current stimulation limits melatonin‐induced vasocontraction. Counter‐regulatory role for BK current depends on PLC‐dependent signaling cross‐talk. Stimulation of BK current involves melatonin‐induced IP_3_‐mediated calcium release leading to activation of BK channels. Stimulation of BK current involves melatonin‐induced activation of PKC leading to inhibition of BK channels. Net effect of both pathways results in hyperpolarization and limitation of vasocontraction.

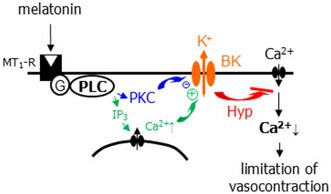

## 1 Introduction

It has been shown that the vascular smooth muscle large‐conductance, calcium‐activated potassium channel (BK channel) is involved in setting the level of spontaneous tone and contributes to various contractile and relaxing responses in different blood vessels. The ability of the BK channel to participate in such diverse processes is due to the fact that the activity of this channel is regulated by a large number of factors, such as the membrane potential and intracellular calcium [1], intracellular pH [2], fatty acids such as omega‐3 fatty acids [3] and several protein kinases (for review see [4]), e.g., PKA [5], PKG [6], and PKC [7]. Under physiological conditions, several, if not all, of these factors act simultaneously, albeit to a different degree, on the BK channel, as they are components of signaling cascades stimulated by external influences such as pressure, neurotransmitters, tissue metabolites, hormones, etc., which continuously act on vessels in vivo. However, little is known about how the cross‐talk of these factors is orchestrated, even for the simple example of PLC‐coupled agonists, which simultaneously stimulate calcium release from intracellular stores, activating the BK channel, and activate PKC, inhibiting the BK channel.

The hormone melatonin is secreted by the pineal gland in high concentrations at night and in very low concentrations during the day. Endogenous melatonin thus informs the organism about the time of day and, due to seasonal changes in the length of the night, also about the seasons. Not surprisingly, endogenous melatonin has been suggested to play an important role in regulating light‐dependent processes and synchronizing different circadian and seasonal rhythms. In addition, exogenous melatonin induces phase shifts in circadian rhythms, which explains its successful clinical use in the treatment of, for example, jetlag and sleep disorders [8, 9]. Furthermore, melatonin has been proposed as an anti‐hypertensive agent [10], in particular during pregnancy [11].

Given the fact that a number of cardiovascular parameters exhibit pronounced circadian rhythms, it is an intriguing finding that receptors for melatonin have been found in the arteries of mice, rats, and humans [12, 13, 14, 15, 16, 17, 18, 19, 20]. In addition, melatonin has been reported to cause contraction [12, 13, 14, 15, 21, 22] or dilation [20, 23, 24] of different vessels, depending on the tissue studied. With regard to the above‐mentioned possible cross‐talk between the branches of the PLC signaling pathway that influences BK channel activity, it should be noted that the vasoactive effects of melatonin have been reported to involve both BK channels [13, 15, 20, 25, 26] as well as the PLC signaling cascade [20, 27, 28]. However, it is not known whether the melatonin‐induced effect on the BK channel is mediated by a coincident stimulation of calcium release from intracellular stores, which can activate the BK channel, and activation of PKC, which can inhibit the BK channel, and if this coincident regulation exists, what its net effect is.

Thus, the hypothesis that cross‐talk of PLC‐mediated stimulation of PKC and of calcium release from intracellular stores determines the role of the BK channel in melatonin‐induced vasocontraction was tested.

## 2 Material and Methods

### 2.1 Vessel Dissection

Male Wistar‐Kyoto rats were housed under standardized conditions at constant 22°C room temperature and a regulated 12‐h light–dark cycle. Rats always had free access to standard pellet chow and drinking water. The age of the rats on the experimental day ranged from 9 to 14 weeks. The animals were killed under CO_2_ narcosis by decapitation according to ARRIVE guidelines; all procedures were approved by the local Animal Care and Use Committee. The main ventral tail artery was then dissected and used for myography and patch‐clamp studies.

### 2.2 Isometric Vessel Preparations

A piece of the artery was threaded onto two 40 μm diameter stainless steel wires and mounted on a wire myograph (model 300A, JP Trading, Denmark) containing physiological salt solution (PSS) consisting of (in mmol l^−1^): 120 NaCl, 4.5 KCl, 1.2 NaH_2_PO_4_, 1.0 MgSO_4_, 1.6 CaCl_2_, 0.025 EDTA, 5.5 glucose, 26 NaHCO_3_, 5 HEPES at pH 7.4, which was continuously bubbled with carbogen (95% O_2_ + 5% CO_2_). Isometric tension was recorded with the program Myodaq (JP Trading, Denmark). After the temperature reached 37.0°C ± 0.5°C, the vessels were stretched radially to their optimal lumen diameter d_o_ corresponding to 90% of the passive diameter of the vessel at 100 mmHg and were allowed to stabilize for 15 min [29]. The endothelium was removed using a rat whisker. Vessel reactivity was tested with three applications of a solution containing 10 μmol l^−1^ noradrenaline. Successful functional removal of the endothelium was confirmed by the absence of a response to 1 μmol l^−1^ acetylcholine after pre‐constriction with 10 μmol l^−1^ noradrenaline. All drugs were applied directly into the experimental chamber.

### 2.3 Cell Isolation

A piece of the artery was placed into a microtube containing 1 mL of an enzyme solution consisting of (in mmol l^−1^): 110 NaCl, 5 KCl, 0.16 CaCl_2_, 2 MgCl_2_, 10 HEPES, 10 NaHCO_3_, 0.5 KH_2_PO_4_, 0.5 NaH_2_PO_4_, 10 glucose, 0.49 EDTA, and 10 taurine at pH 7.0, as well as 1.5 mg ml^−1^ papain, 1.6 mg ml^−1^ albumin, and 0.4 mg ml^−1^ DL‐dithiothreitol and stored there overnight at 4°C. The next day, the microtube with the vessel was incubated for 5–20 min at 37°C. Single cells were released by trituration with a polyethylene pipette. The following solutions have been used:

1. bath solution: 135 NaCl, 6 KCl, 1 MgCl_2_, 0.1 CaCl_2_, and 10 HEPES (in mmol l^−1^) at pH 7.4.
2. pipette solution: 105 KCl, 10 NaCl, 1 MgCl_2_, 10 BAPTA, 0.1 ATP, 10 HEPES (in mmol l^−1^) and an appropriate amount of CaCl_2_ to get a [Ca]_i_ of 100 nmol l^−1^ at pH 7.4 [30].

### 2.4 Patch‐Clamp Recording

Patch pipettes had resistances of 2–3 MΩ. Recordings were made with an Axopatch 200 amplifier with the whole‐cell patch‐clamp configuration. In these experiments, stimulation of currents with pulse protocols, data sampling at a rate of 1 kHz, and data analysis were done with the software package ISO2 (MFK, Germany). Stability of the access and leak resistance was tested regularly during the course of the experiment; non‐stable recordings were discarded. All experiments were performed at room temperature.

### 2.5 Drugs and Chemicals

Albumin, DL‐dithiothreitol, TEA, 4‐aminopyridine, melatonin, heparin, and the salts for the solutions were obtained from Sigma. Papain was from Ferak (Berlin, Germany). Calphostin C, SQ 22536, GDPβS, U‐73122, and U‐73343 were purchased from Calbiochem. Luzindole and 4P‐PDOT were from TOCRIS. Iberiotoxin was obtained from Alomone.

### 2.6 Statistics

All data are presented as mean ± SEM, n is the number of vessels or cells, respectively. Statistical analysis was performed using repeated measures ANOVA, one‐way ANOVA, paired samples and independent samples *t*‐test as appropriate (SPSS 9.0 for Windows), *p* < 0.05 was considered statistically significant.

## 3 Results

### 3.1 Melatonin Stimulates Outward Currents

**FIGURE 1:**
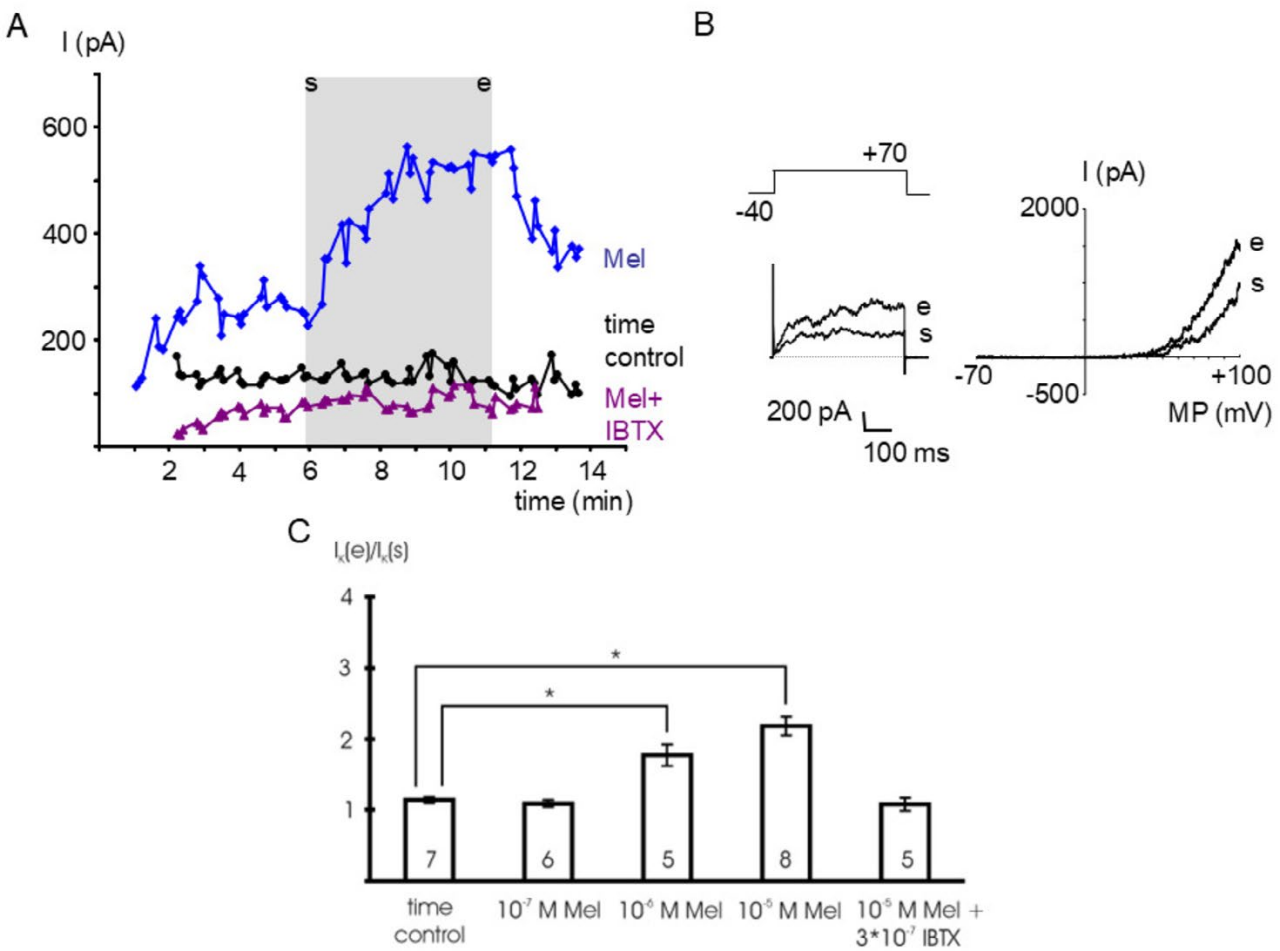
Melatonin stimulates outward currents. (A) time course of the outward current at a test potential of +70 mV during the addition of 10^−5^ mol l^−1^ melatonin (Mel), of bath solution (time control) and of 10^−5^ mol l^−1^ melatonin in the presence of 3*10^−7^ mol l^−1^ iberiotoxin, a specific BK channel inhibitor (Mel + IBTX). The application period of melatonin and of the bath solution, respectively, is indicated by the gray area; s—start of application, e—end of application; data points are the mean current during the last 200 ms of the voltage step. (B) Example traces of the outward current at the start (s) and the end (e) of melatonin application evoked by a voltage step from a holding potential of −40 mV to a test potential of +70 mV with duration 500 ms (left panel) and a voltage ramp from −70 mV to +100 mV providing the I‐V relationship (right panel), respectively. (C) Summarized data of the effect of melatonin (Mel) on the outward current, data are expressed as the ratio of the current at the end of the application period (I_K_ (e)) to the initial current immediately at the start of the application period (I_K_ (s)); test potential +70 mV; number of cells investigated appears on bar; * ‐ *p* < 0.05.

In isolated single smooth muscle cells, melatonin reversibly augmented an outward current over a wide range of membrane potentials (Figure 1A,B). The effect of melatonin was concentration‐dependent (Figure 1C): 10^−6^ mol l^−1^ and 10^−5^ mol l^−1^ melatonin stimulated the current at +70 mV compared to vehicle application during the time control (*p* < 0.05). In contrast, in the presence of 3*10^−7^ mol l^−1^ iberiotoxin, a specific BK channel inhibitor [31], we did not detect any effect of 10^−5^ mol l^−1^ melatonin on the outward current compared to the time control (*p* = 0.98) (Figure 1A,C).

### 3.2 The Effect of Melatonin Is Mediated by Melatonin Receptors

**FIGURE 2:**
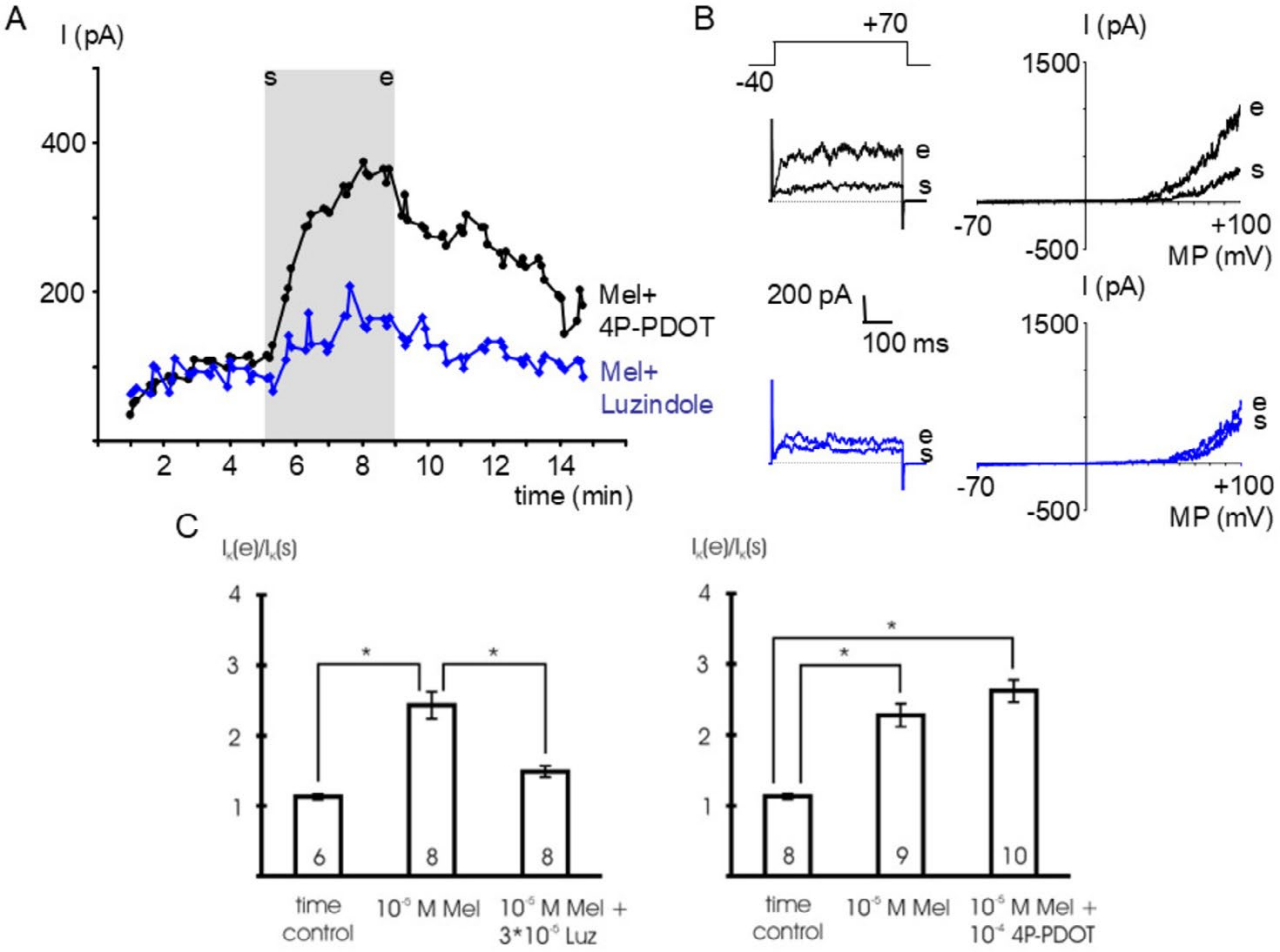
The effect of melatonin is mediated by melatonin‐receptors. (A) time course of the outward current at a test potential of +70 mV during the addition of 10^−5^ mol l^−1^ melatonin in the presence of 10^−4^ mol l^−1^ 4P‐PDOT, a selective MT_2_–receptor antagonist (Mel+4P‐PDOT), and of 10^−5^ mol l^−1^ melatonin in the presence of 3*10^−5^ mol l^−1^ luzindole, a MT_1_‐ and MT_2_‐receptor antagonist (Mel+Luzindole). The application period of melatonin is indicated by the gray area; s—start of application, e—end of application; data points are the mean current during the last 200 ms of the voltage step. (B) Example traces of the outward current at the start (s) and the end (e) of melatonin application evoked by a voltage step from a holding potential of −40 mV to a test potential of +70 mV with duration 500 ms (left panels) and a voltage ramp from −70 mV to +100 mV providing the I‐V relationship (right panels), respectively. Upper panels—in the presence of 4P‐PDOT, lower panels—in the presence of luzindole. (C) Summarized data of the effect of melatonin (Mel) in the presence of luzindole (Luz) and of 4P‐PDOT, respectively on the outward current; data are expressed as the ratio of the current at the end of the application period (I_K_ (e)) to the initial current immediately at the start of the application period (I_K_ (s)); test potential +70 mV; number of cells investigated appears on bar; * ‐ *p* < 0.05.

In the presence of 10^−4^ mol l^−1^ 4P‐PDOT, a selective MT_2_‐receptor antagonist [28, 32], 10^−5^ mol l^−1^ melatonin stimulated the BK current at +70 mV; we did not detect any difference between the effect of melatonin in the presence and absence of 4P‐PDOT (*p* = 0.25) (Figure 2A–C). In contrast, in the presence of 3*10^−5^ mol l^−1^ luzindole, a MT_1_‐ and MT_2_‐receptor antagonist [28, 32], we did not observe any effect of 10^−5^ mol l^−1^ melatonin on the BK current at +70 mV compared to the time control (*p* = 0.23) (Figure 2A–C).

### 3.3 The Effect of Melatonin Is Mediated by G‐Proteins and PLC

**FIGURE 3:**
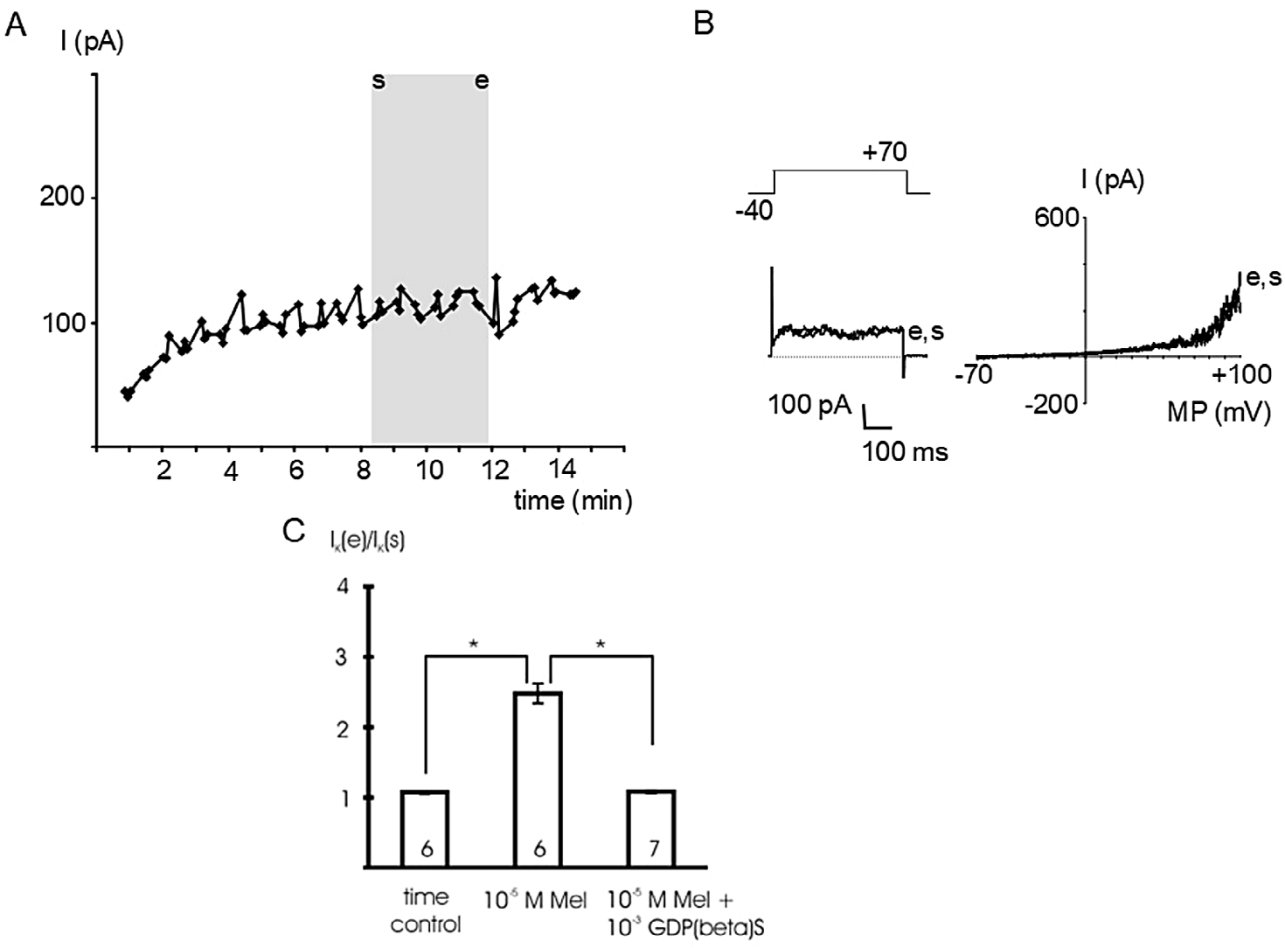
The effect of melatonin is mediated by G‐proteins. (A) time course of the outward current at a test potential of +70 mV during the addition of 10^−5^ mol l^−1^ melatonin after a 6–7 min infusion of 10^−3^ mol l^−1^ GDPβS, an inhibitor of G‐protein function, via the patch pipette into the cell. The application period of melatonin is indicated by the gray area; s—start of application, e—end of application; data points are the mean current during the last 200 ms of the voltage step. (B) Example traces of the outward current at the start (s) and the end (e) of melatonin application evoked by a voltage step from a holding potential of −40 mV to a test potential of +70 mV with duration 500 ms (left panel) and a voltage ramp from −70 mV to +100 mV providing the I‐V relationship (right panel), respectively. (C) Summarized data of the effect of melatonin (Mel) in the presence of GDPβS on the outward current, data are expressed as the ratio of the current at the end of the application period (I_K_ (e)) to the initial current immediately at the start of the application period (I_K_ (s)); test potential +70 mV; number of cells investigated appears on bar; * ‐ *p* < 0.05.

After a 6–7 min infusion of 10^−3^ mol l^−1^ GDPβS, an inhibitor of G‐protein function, via the patch pipette into the cell, we did not observe any effect of 10^−5^ mol l^−1^ melatonin on the BK current at +70 mV compared to the time control (*p* = 1.00) (Figure 3A–C).

**FIGURE 4:**
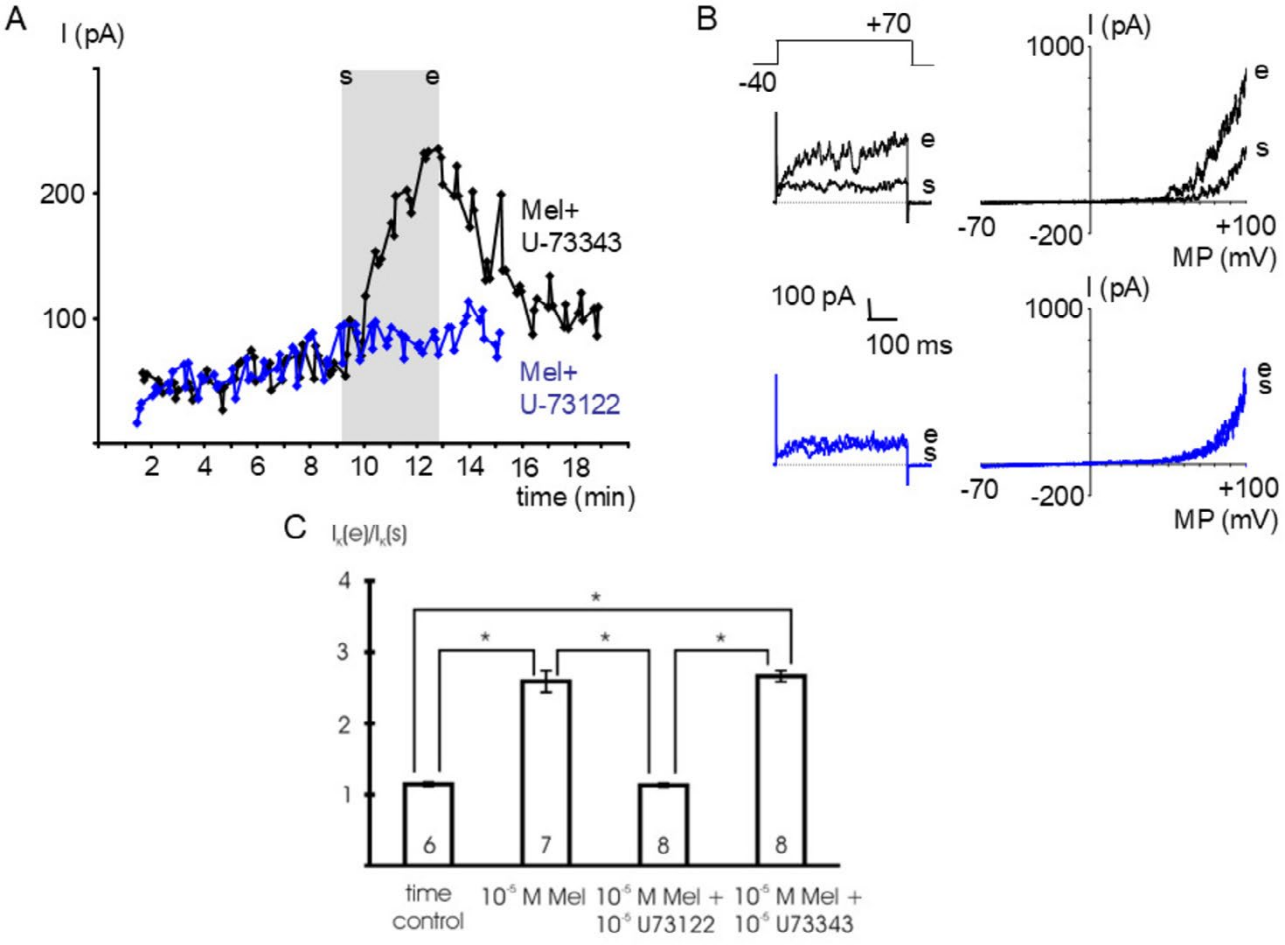
The effect of melatonin is mediated by PLC. (A) time course of the outward current at a test potential of +70 mV during the addition of 10^−5^ mol l^−1^ melatonin during the intracellular presence of 10^−5^ mol l^−1^ U‐73122, a PLC‐inhibitor (Mel+U‐73122) and of 10^−5^ mol l^−1^ melatonin during the intracellular presence of 10^−5^ mol l^−1^ U‐73343, an inactive analogue of the PLC‐inhibitor U‐73122 (Mel+U‐73343). The application period of melatonin is indicated by the gray area; s—start of application, e—end of application; data points are the mean current during the last 200 ms of the voltage step. (B) Example traces of the outward current at the start (s) and the end (e) of melatonin application evoked by a voltage step from a holding potential of −40 mV to a test potential of +70 mV with duration 500 ms (left panel) and a voltage ramp from −70 mV to +100 mV providing the I‐V relationship (right panel), respectively. Upper panels—in the presence of U‐73343, lower panels‐in the presence of U‐73122. (C) Summarized data of the effect of melatonin (Mel) in the presence of U‐73122 and U‐73343 on the outward current, data are expressed as the ratio of the current at the end of the application period (I_K_ (e)) to the initial current immediately at the start of the application period (I_K_ (s)); test potential +70 mV; number of cells investigated appears on bar; * *‐ p* < 0.05.

In the presence of 10^−5^ mol l^−1^ U‐73122, a PLC‐inhibitor [33], we did not detect any effect of 10^−5^ mol l^−1^ melatonin on the BK current at +70 mV compared to the time control (*p* = 1.00) (Figure 4A–C). In contrast, in the presence of 10^−5^ mol l^−1^ U‐73343, an inactive analogue of the PLC‐inhibitor U‐73122 [33], 10^−5^ mol l^−1^ melatonin stimulated the BK current at +70 mV; we did not observe any difference between the effect of melatonin in the presence and absence of U‐73343 (*p* = 1.00) (Figure 4A–C). Both U‐73122 and U‐73343 were infused into the cells via the patch pipette because bath application of U‐73122 induced an inhibition of the BK current accompanied by an increase in current noise. Application of the inhibitors alone via the patch pipette did not alter the BK current (data not shown).

### 3.4 The Effect of Melatonin Involves Release of Calcium From IP_3_ ‐Sensitive Stores

**FIGURE 5:**
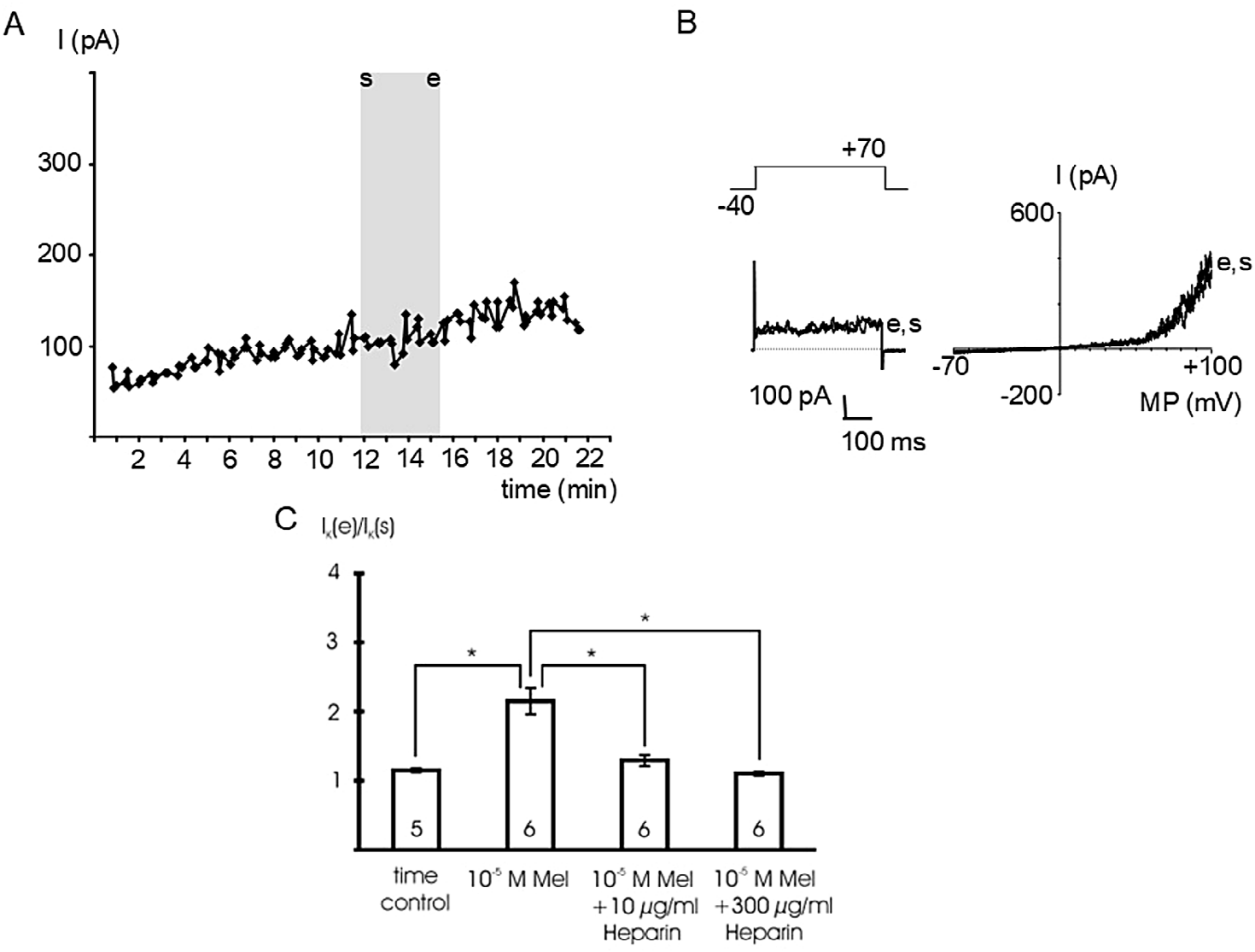
The effect of melatonin involves release of calcium from IP_3_‐sensitive stores. (A) time course of the outward current at a test potential of +70 mV during the addition of 10^−5^ mol l^−1^ melatonin after a 10–11 min infusion of 300 μg ml^−1^ low molecular weight heparin, an inhibitor of IP_3_ –induced calcium release, via the patch pipette. The application period of melatonin is indicated by the gray area; s—start of application, e—end of application; data points are the mean current during the last 200 ms of the voltage step. (B) Example traces of the outward current at the start (s) and the end (e) of melatonin application evoked by a voltage step from a holding potential of −40 mV to a test potential of +70 mV with duration 500 ms (left panel) and a voltage ramp from −70 mV to +100 mV providing the I‐V relationship (right panel), respectively. (C) Summarized data of the effect of melatonin (Mel) in the presence of heparin on the outward current, data are expressed as the ratio of the current at the end of the application period (I_K_ (e)) to the initial current immediately at the start of the application period (I_K_ (s)); test potential +70 mV; number of cells investigated appears on bar; * ‐ *p* < 0.05.

After a 10–11 min infusion of 300 μg ml^−1^ low molecular weight heparin, an inhibitor of IP_3_ –induced calcium release [34], via the patch pipette, we did not observe any effect of 10^−5^ mol l^−1^ melatonin on the BK current at +70 mV compared to the time control (*p* = 1.00) (Figure 5A–C).

### 3.5 The Effect of Melatonin Involves Activation of PKC

**FIGURE 6:**
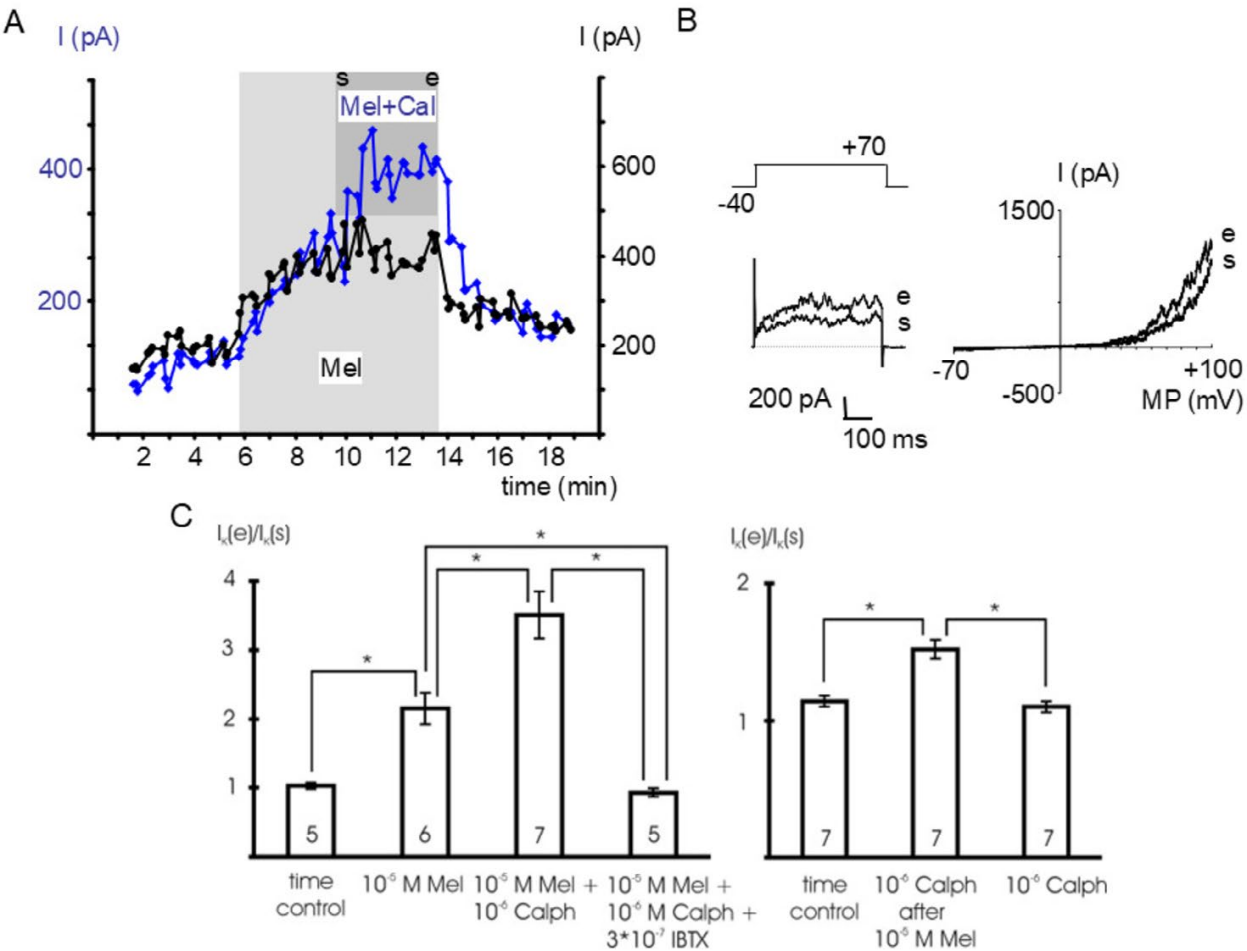
The effect of melatonin involves activation of PKC. (A) time course of the outward current at a test potential of +70 mV during the addition of 10^−6^ mol l^−1^ calphostin C, a specific PKC‐inhibitor, in the presence of 10^−5^ mol l^−1^ melatonin (Mel+Cal) and of 10^−5^ mol l^−1^ melatonin alone (Mel). The application period of calphostin C is indicated by the dark gray area, s—start of application, e—end of application of calphostin C; the application period of melatonin is indicated by the light gray area; data points are the mean current during the last 200 ms of the voltage step. (B) Example traces of the outward current at the start (s) and the end (e) of calphostin C application evoked by a voltage step from a holding potential of −40 mV to a test potential of +70 mV with duration 500 ms (left panel) and a voltage ramp from −70 mV to +100 mV providing the I‐V relationship (right panel), respectively. (C) Summarized data of the effect of calphostin C (Calph) and melatonin (Mel) on the outward current, data are expressed as the ratio of the current at the end of the application period (I_K_ (e)) to the initial current immediately at the start of the application period (I_K_ (s)), I_K_ (s) is the current at the start of melatonin application (left panel) and at the start of calphostin C application (right panel), respectively; test potential +70 mV; number of cells investigated appears on bar; * ‐ *p* < 0.05.

Addition of 10^−6^ mol l^−1^ calphostin C, a specific PKC‐inhibitor [35], in the presence of 10^−5^ mol l^−1^ melatonin increased the BK current at +70 mV (summarized effect of melatonin and calphostin C) compared to the effect of 10^−5^ mol l^−1^ melatonin alone (*p* < 0.05) (Figure 6A–C left panel). Thus, after stimulation by 10^−5^ mol l^−1^ melatonin, calphostin C at 10^−6^ mol l^−1^ additionally increased the BK current at +70 mV (effect of calphostin C alone) compared with vehicle application during time control (*p* < 0.05) (Figure 6A–C right panel). In contrast, in the absence of melatonin, we did not observe any effect of calphostin C at 10^−6^ mol l^−1^ on the BK current at +70 mV compared to the time control (*p* = 1.00) (Figure 6C, right panel). Further, in the presence of 10^−7^ mol l^−1^ iberiotoxin, we did not detect any effect of 10^−6^ mol l^−1^ calphostin C together with 10^−5^ mol l^−1^ melatonin on the BK current at +70 mV compared to the time control (*p* = 1.00) (Figure 6C, left panel).

### 3.6 The Effect of Melatonin Does Not Involve Inhibition of Adenylate Cyclase

**FIGURE 7:**
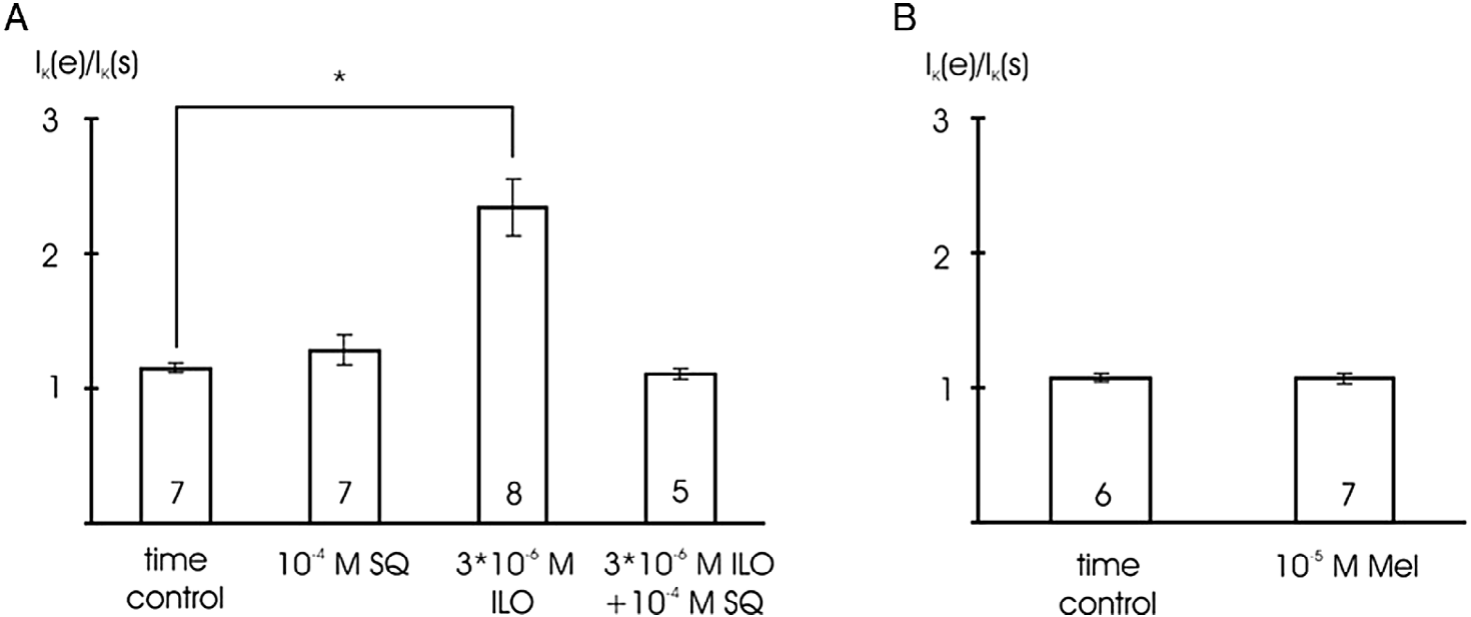
The effect of melatonin does not involve inhibition of adenylate cyclase. (A) Summarized data of the effect of 10^−4^ mol l^−1^ SQ‐22536 (SQ), an adenylate cyclase inhibitor, 3*10^−6^ mol l^−1^ iloprost (ILO) and 3*10^−6^ mol l^−1^ iloprost in the presence of 10^−4^ mol l^−1^ SQ‐22536 (ILO + SQ) on the outward current, data are expressed as the ratio of the current at the end of the application period (I_K_ (e)) to the initial current immediately at the start of the application period (I_K_ (s)); test potential +70 mV; number of cells investigated appears on bar; * ‐ *p* < 0.05; (B) Summarized data of the effect of 10^−5^ mol l^−1^ melatonin (Mel) and of vehicle (time control) after blocking the PLC cascade by infusion of 10^−5^ mol l^−1^ U‐73122 via the patch pipette and after activating the adenylate cyclase cascade by 3*10^−6^ mol l^−1^ iloprost on the outward current, data are expressed as the ratio of the current at the end of the application period (I_K_ (e)) to the initial current immediately at the start of the application period (I_K_ (s)); test potential +70 mV; number of cells investigated appears on bar.

Addition of 3*10^−6^ mol l^−1^ iloprost stimulated the BK current at +70 mV compared to vehicle application during the time control (*p* < 0.05). In contrast, in the presence of 10^−4^ mol l^−1^ SQ‐22536, an adenylate cyclase inhibitor [36], we did not detect any effect of 3*10^−6^ mol l^−1^ iloprost on the BK current at +70 mV compared to the time control (*p* = 1.00). Further, we did not observe any effect of 10^−4^ mol l^−1^ SQ‐22536 alone, on the BK current at +70 mV compared to the time control (*p* = 0.85) (Figure 7A). In addition, after infusion of 10^−5^ mol l^−1^ U‐73122 via the patch pipette in the presence of 3*10^−6^ mol l^−1^ iloprost, we did not observe any effect of 10^−5^ mol l^−1^ melatonin on the BK current at +70 mV compared to the time control (*p* = 0.91) (Figure 7B).

### 3.7 The Melatonin‐Induced Activation of BK Channels Limits Melatonin‐Induced Contractions

**FIGURE 8:**
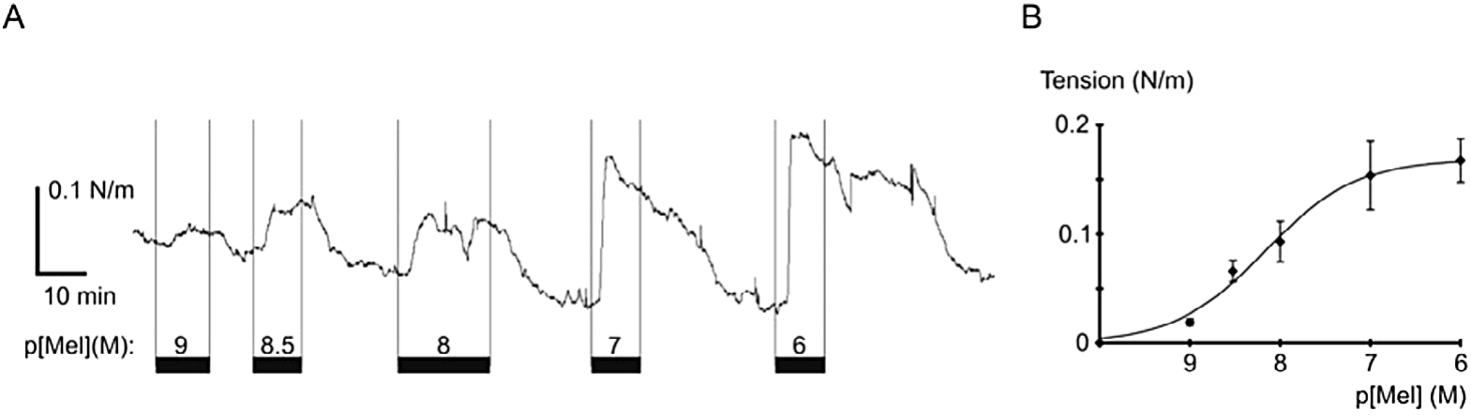
Melatonin contracts the artery. (A) Example trace of vessel tension during application of melatonin (Mel) at different concentrations; application of Mel is indicated by horizontal bars. (B) Melatonin concentration‐response relationship was fitted with the equation y = y_max_*(c^n^/(c^n^ + EC_50_^n^)).

Application of melatonin increased vessel tension resulting in a concentration‐dependent reversible contraction (Figure 8A). Half‐maximal contraction occurred at an EC_50_ of 6.8*10^−9^ mol l^−1^ melatonin. The concentration‐response curve was further characterized by a slope factor of *n* = 0.87 and a maximum reaching 0.17 N/m (Figure 8B).

**FIGURE 9:**
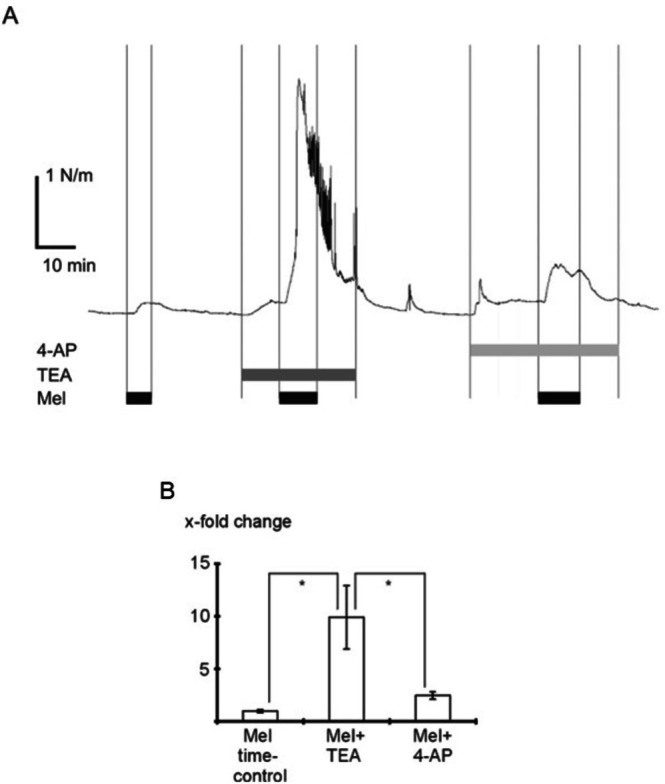
The melatonin‐induced activation of BK channels limits the melatonin‐induced contraction. Example trace of vessel tension during application of 10^−6^ mol l^−1^ melatonin alone (Mel time control) and in the presence of 10^−3^ mol l^−1^ TEA, a selective inhibitor of BK channels at this concentration or 5*10^−4^ mol l^−1^ 4‐AP, an inhibitor of voltage‐gated potassium channels; application of the substances is indicated by horizontal bars. (B) Summarized data of the effect of melatonin alone (Mel), melatonin in the presence of TEA and melatonin in the presence of 4‐AP on vessel tension, data are expressed as the x‐fold change in tension compared to an initial application of melatonin prior to the Mel time control or melatonin application in the presence of TEA / 4‐AP; *n* = 7; * ‐ *p* < 0.05.

In the presence of 10^−3^ mol l^−1^ TEA, a selective inhibitor of BK channels at this concentration [1], 10^−6^ mol l^−1^ melatonin increased vessel tension considerably compared to the time control addition of 10^−6^ mol l^−1^ melatonin alone (*p* < 0.05) (Figure 9A,B). TEA itself increased vessel tension (Figure 9A). Thus, the alteration of the effect of melatonin in the presence of TEA could be due to the TEA‐induced change in the initial contractile state of the vessel rather than the TEA‐evoked inhibition of the BK channel. However, the increase in vessel tension observed after application of 10^−6^ mol l^−1^ melatonin in the presence of 10^−3^ mol l^−1^ TEA was different from the effect of 10^−6^ mol l^−1^ melatonin in the presence of 5*10^−4^ mol l^−1^ 4‐AP, an inhibitor of voltage‐gated potassium channels [1] (*p* < 0.05) (Figure 9A,B). The concentration of 4‐AP was matched to produce an effect not different from the action of 10^−3^ mol l^−1^ TEA. Moreover, in the presence of 10^−7^ mol l^−1^ iberiotoxin, a highly selective inhibitor of BK channels [31], 10^−6^ mol l^−1^ melatonin increased vessel tension 15.7 ± 4.4‐fold (*n* = 7) compared to the time control addition of melatonin alone (0.92 ± 0.09‐fold, *n* = 7; *p* < 0.05).

## 4 Discussion

We found that melatonin markedly stimulates outward currents in freshly isolated smooth muscle cells from rat tail arteries; this stimulation was eliminated in the presence of IBTX. The effect of melatonin on the IBTX‐sensitive outward current was abolished by luzindole but was not affected by 4P‐PDOT. Mechanistically, GDPβS, low molecular weight heparin, and U‐73122, but not U‐73343, inhibited the effect of melatonin on the IBTX‐sensitive outward current. Calphostin C enhanced the effect of melatonin on the IBTX‐sensitive current, suggesting functional cross‐talk between distinct PLC‐dependent signaling branches. Under conditions of blocked PLC‐ and activated cAMP‐signaling, melatonin had no effect on the IBTX‐sensitive outward current. At the functional level, melatonin induced a concentration‐dependent contraction in intact rat tail arteries. This contraction was augmented in the presence of TEA and iberiotoxin but not in the presence of 4‐AP.

### 4.1 Melatonin Stimulates BK Currents

The results of the present study demonstrate that melatonin is capable of enhancing outward currents in freshly isolated single smooth muscle cells from rat tail artery. Under the experimental conditions used in the present study, the outward current is mainly carried by BK channels [37]. In fact, the melatonin‐induced effect was abolished by iberiotoxin, a specific inhibitor of BK channels [31]. This suggests that melatonin affects BK currents. This finding is supported by the observation that melatonin stimulates BK currents in rat cerebral artery smooth muscle cells [20].

### 4.2 The Melatonin‐Induced Stimulation of BK Currents Is Mediated by MT_1_ ‐Melatonin Receptors

The melatonin‐induced stimulation of the BK current was not affected by 4P‐PDOT, a selective MT_2_–receptor antagonist, but was considerably depressed by luzindole, a MT_1_‐ and MT_2_–receptor antagonist [28], suggesting that the effect observed in the present study is mediated by MT_1_‐receptors. This conclusion is supported by the finding that melatonin‐induced contractions of wire‐mounted preparations of rat tail arteries, the same vessels as explored in the present study, are mediated by MT_1_ receptors [25]. Furthermore, luzindole has been shown to inhibit melatonin‐induced contractions in rat middle cerebral arteries in vitro [13] and rat cerebral arterioles in vivo [15], as well as the melatonin‐induced activation of BK currents and BK channels in rat cerebral artery smooth muscle cells [20]. The latter findings are consistent with melatonin acting via MT_1_‐receptors, although the experimental designs did not exclude participation of MT_2_‐receptors. Furthermore, MT_1_ receptor mRNA, but not MT_2_ receptor mRNA, was detected in rat tail arteries [16], and the cumulated evidence summarized in a recent review [38] suggests that melatonin‐induced vasocontraction, as for example observed in the present study (see below), is mediated by melatonin MT_1_ receptors, while melatonin‐induced vasorelaxation is mediated by melatonin MT_2_ receptors.

### 4.3 The Melatonin‐Induced Stimulation of BK Currents Is Mediated by PLC

The melatonin‐induced stimulation of the BK current was abolished by GDPβS, an inhibitor of G‐protein function, and by U‐73122, a PLC inhibitor. In contrast, the melatonin‐induced stimulation of the BK current was not affected by U‐73343, an inactive analogue of U‐73122, providing evidence for a specific action of the PLC inhibitor U‐73122. This finding is supported by the observation that the melatonin‐induced stimulation of BK currents and of single BK channels in rat cerebral artery smooth muscle cells was also inhibited by U‐73122, but not by U‐73343 [20]. In general, melatonin receptors have been shown to be coupled to the PLC cascade [27, 28].

### 4.4 The Melatonin‐Induced Stimulation of BK Currents Involves IP_3_ ‐Induced Calcium Release

The melatonin‐induced stimulation of the BK current was abolished by low molecular weight heparin, an inhibitor of IP_3_ –induced calcium release. This finding is consistent with earlier reports of BK current activation by histamine‐induced calcium release in rabbit coronary artery smooth muscle cells [39] and the inhibition of noradrenaline‐induced stimulation of BK currents by heparin in rabbit portal vein smooth muscle cells [40]. Along with IP_3_‐induced calcium release, calcium sparks are an important component of BK channel activity, for example in cerebral and coronary arteries [41, 42, 43]. In other vessels, however, BK channel activity is independent of calcium sparks [44, 45], in particular in the rat tail arteries used in the present study [46]. Therefore, it seems unlikely that calcium sparks are involved in the regulation of BK channel activity by melatonin.

### 4.5 The Melatonin‐Induced Stimulation of BK Currents Involves Activation of PKC

The data from the present study show that calphostin C, a specific PKC inhibitor, increases the BK current in the presence of melatonin. The latter effect does not appear to be due to a PKC‐independent interaction of calphostin C with the BK current, as calphostin C did not affect the BK current in the absence of melatonin. In contrast, the PKC inhibitor Ro31–8220 inhibited the melatonin‐induced stimulation of BK currents and BK channels in rat cerebral artery smooth muscle cells [20]. Of note, melatonin induced vasodilation in this vessel [20] rather than vasocontraction, as observed in rat tail arteries in the present study, suggesting vessel‐specific differences in melatonin signaling pathways.

In vascular smooth muscle, PKC has been shown to affect not only the BK channel [47], but also voltage‐dependent [48] and ATP‐sensitive [49] potassium channels. However, in the present study, iberiotoxin completely inhibited the effect of calphostin C, indicating that the effect of calphostin C was mediated solely by BK channels. Indeed, it has been reported that BK channels in vascular smooth muscle are affected by PKC. Thus, it has been shown that specific PKC isozymes activate BK channels in smooth muscle cells from rat pulmonary arteries [50]. However, in most studies, i.e., in smooth muscle cells from porcine coronary arteries [7], from rat mesenteric arteries [51] and from rat tail arteries, the vessel explored in the present study [47], PKC has been shown to inhibit BK channel activity. These reports and the data from the present study suggest that PKC partly inhibits the BK current during melatonin application.

With regard to the contribution of PKC to the melatonin‐induced stimulation of the BK current, we observed that the PKC‐inhibitor calphostin C affected the BK current in the presence of melatonin but not in its absence. This suggests that PKC is active in the presence of melatonin but not in its absence, indicating an activation of PKC by melatonin. In summary, the data from the present study show that the melatonin‐induced stimulation of the BK current involves melatonin‐induced activation of PKC leading to a partial reduction of the BK current.

Of note, the adenylate cyclase cascade was suggested to mediate the potentiating effect of melatonin on vasocontraction in rat tail arteries [25]. The present study showed that iloprost stimulates the BK current and that the adenylate cyclase inhibitor SQ‐22536 abolished this effect. Furthermore, a previous study on the same cells showed that PKA inhibitors prevent the iloprost‐induced stimulation of the BK current [37]. Neither PKA inhibitors [37] nor the adenylate cyclase inhibitor SQ‐22536 used in the present study had any effect on the BK current on their own. Thus, the cAMP pathway was not active in the absence of iloprost but iloprost activates the cAMP pathway coupled to the BK channel. It is noteworthy that melatonin did not affect the BK current when the cAMP signaling pathway was activated by iloprost and the PLC signaling pathway was inhibited by U‐73122 to eliminate interference from the PLC signaling pathway. This finding is supported by the observation that melatonin did not affect cAMP accumulation in rat tail arteries [16]. Thus, the data from the present study do not provide evidence for a contribution of cAMP signaling in the melatonin‐induced stimulation of the BK current.

### 4.6 The Melatonin‐Induced Activation of BK Channels Limits Melatonin‐Induced Contractions

It has been found that physiological serum levels of melatonin are approximately 10^−9^ M (during the night) [52]. Interestingly, concentrations up to 10^−5^ M are achieved with pharmacological application [52]. The results of the present study demonstrate for the first time that melatonin contracts rat tail arteries with an EC_50_ of 6.8*10^−9^ mol l^−1^. A direct contractile effect of melatonin with similar EC_50_ values has been observed previously in cerebral arteries [13, 15]. However, earlier studies on rat tail arteries reported that melatonin has no direct contractile effect, except in vessels of juvenile animals [53], but rather potentiated the effect of vasoconstrictors or electrical field stimulation, with EC_50_ values similar to those observed for its direct vasocontractile effect in the present study, e.g., [12, 21, 25, 54, 55]. It should be noted that the results of the present study show that melatonin can induce clear contractions of the rat tail artery (see Figure 8), although these contractions were relatively small compared to other agonists and required a very careful preparation of the vessel. Nevertheless, the reason for the discrepancy between the observations of the present study and earlier reports is unclear.

The effect of melatonin was considerably augmented in the presence of TEA at a concentration specific for BK channels [1]. TEA itself increased vessel tension. Thus, the augmentation of the effect of melatonin in the presence of TEA could be attributed to the TEA‐induced change in the initial contractile state of the vessel rather than to the TEA‐induced inhibition of the BK channel. To distinguish between these two possibilities, a similar change in the initial contractile state was induced by 4‐AP, an inhibitor of voltage‐gated potassium channels [1]. This altered the initial contractile state of the vessel by the same mechanisms, with the exception of the very first element of the signaling cascade (voltage‐dependent potassium channels in the presence of 4‐AP instead of BK channels in the presence of TEA). Importantly, melatonin induced a much larger contraction in the presence of TEA than in the presence of 4‐AP. Furthermore, a considerably increased melatonin‐induced contraction was also observed in the presence of iberiotoxin, a specific inhibitor of BK channels [31]. The inhibition of BK channels thus unmasks a large contractile effect of melatonin in rat tail arteries. However, if BK channels are available, melatonin‐induced contractions are smaller due to the relaxing effect of BK channels. In detail, the presented data show that the melatonin‐induced BK channel activation occurs via a dual process. First, melatonin induces calcium release‐mediated activation of BK channels, which itself has a dilating effect impeding melatonin‐induced vasocontraction. Second, melatonin induces a PKC‐mediated inhibition of BK channels, which itself facilitates melatonin‐induced vasocontraction. Since the PKC‐mediated inhibition of BK channels merely reduces, but does not override, the calcium release‐mediated activation of BK channels, the net effect is a stimulation of the BK current. BK channels therefore limit the melatonin‐induced contraction of the vessel.

Many contractile agonists activate the PLC‐pathway, which is coupled to calcium release and PKC activation. Thus, it is conceivable that the melatonin‐induced PLC‐dependent signaling cross‐talk between intracellular calcium and PKC, which affects the BK channel, is also operating for other contractile agonists. To the best of our knowledge, however, this assumption is not currently supported by studies on other contractile agonists and is therefore rather speculative.

The mechanisms by which melatonin can affect BK channel activity to regulate vascular tone appear to be complex. For example, BK channel activity in the tail artery is independent of calcium sparks [46], whereas calcium sparks are an important component of BK channel activity in cerebral arteries [41, 42, 43]. In the latter arteries, multiple mechanisms have been suggested to mediate the effect of melatonin on BK channels, including calcium spark regulation by the PLC‐PKC pathway [20]. Moreover, indirect evidence from functional studies on intact vessel preparations suggests that melatonin can inhibit BK channels [13, 15], most likely mediated by G_i_‐proteins, i.e., by cAMP signaling [13]. Thus, the precise mechanism by which melatonin affects BK channel activity to regulate vascular tone appears to depend on vessel‐specific signaling pathways. A transfer of our findings to other vascular beds will only be possible once these vessel specific, melatonin‐activated signaling pathways have been identified.

In conclusion, the novel observation of this study is that BK currents from rat tail artery smooth muscle cells are stimulated by a melatonin‐induced specific orchestration of PKC‐mediated inhibition and calcium release‐mediated activation of BK channels. The net effect is a stimulation of the BK current. This BK channel stimulation limits melatonin‐induced vasocontraction, indicating a counter‐regulatory role for BK channels.

## Author Contributions

Anastasia Pyanova, Vjatscheslav U. Kalentchuk, Vladimir N. Serebryakov, and Rudolf Schubert conceived and designed the research; Anastasia Pyanova, Vjatscheslav U. Kalentchuk, Vladimir N. Serebryakov performed the research and acquired the data; Anastasia Pyanova, Vjatscheslav U. Kalentchuk, Vladimir N. Serebryakov, and Rudolf Schubert analyzed and interpreted the data; all authors were involved in drafting and revising the manuscript.

## Funding

The authors have nothing to report.

## Disclosure

The authors have nothing to report.

## Conflicts of Interest

The authors declare no conflicts of interest.

## Data Availability

The data that support the findings of this study are available in the Materials and Methods, Results, and/or Supporting Information of this article.
